# Supplementary material for: Splice-Junction-Based Mapping of Alternative Isoforms in the Human Proteome
Source: Cell Rep. Author manuscript; Available in PMC 2020 Jan 15. (PMC6961840; doi:10.1016/j.celrep.2019.11.026)
Supplement: 2 [file NIHMS1546469-supplement-2.zip › DF1/PXD010154/Liver/Liver_38_IYD_KPLDQIMVTPTGLK.pdf]

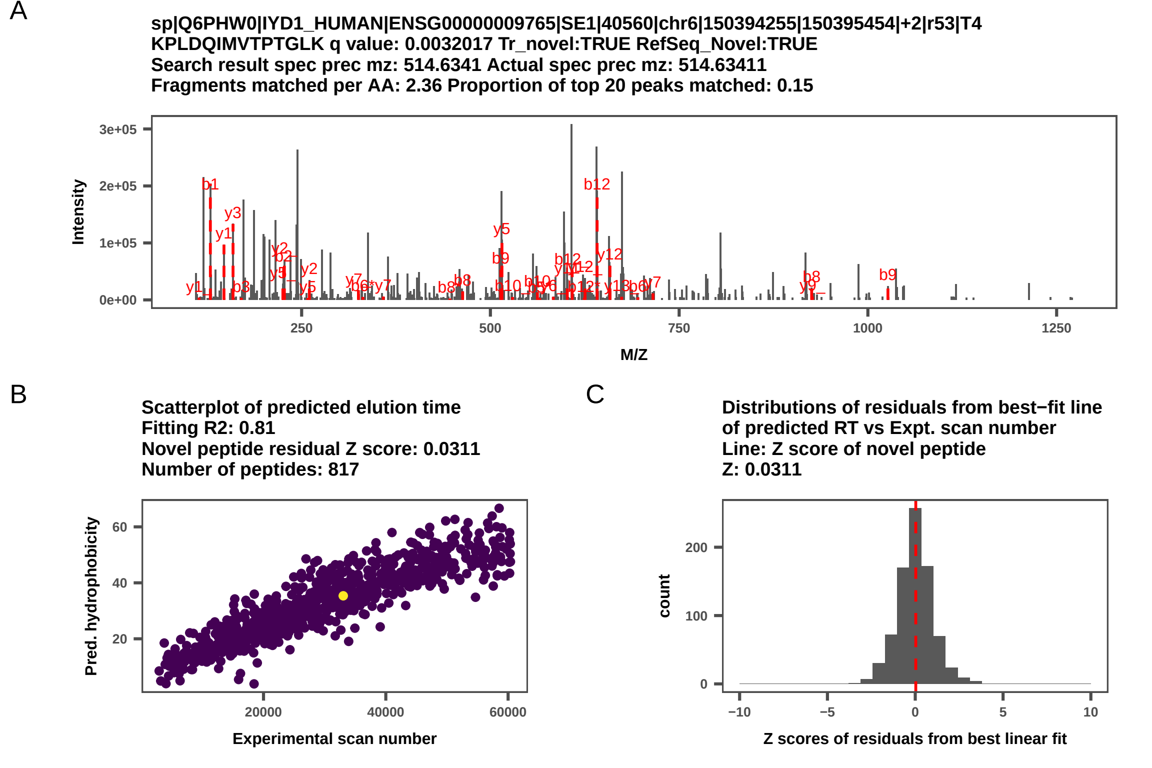
A
sp|Q6PHW0|IYD1_HUMAN|ENSG00000009765|SE1|40560|chr6|150394255|150395454|+2|r53|T4
KPLDQIMVTPTGLK q value: 0.0032017 Tr_novel:TRUE RefSeq_Novel:TRUE
Search result spec prec mz: 514.6341 Actual spec prec mz: 514.63411
Fragments matched per AA: 2.36 Proportion of top 20 peaks matched: 0.15
Intensity
3e+05
2e+05
1e+05
0e+00
b1
y3
y1
y5
b9
b12
y12
b8
b9
250
500
750
1000
1250
M/Z
B
Scatterplot of predicted elution time
Fitting R2: 0.81
Novel peptide residual Z score: 0.0311
Number of peptides: 817
Pred. hydrophobicity
60
40
20
20000
40000
60000
Experimental scan number
C
Distributions of residuals from best−fit line
of predicted RT vs Expt. scan number
Line: Z score of novel peptide
Z: 0.0311
count
200
100
0
−10
−5
0
5
10
Z scores of residuals from best linear fit
